# The knowledge-based products and economic complexity in developing countries

**DOI:** 10.1016/j.heliyon.2019.e02979

**Published:** 2019-12-18

**Authors:** Hamid Sepehrdoust, Razieh Davarikish, Maryam Setarehie

**Affiliations:** a Department of Economics, Bu-Ali-Sina University, Hamedan, Iran; b Faculty of Economics and Social Sciences, Bu-Ali Sina University, Hamadan, Iran

**Keywords:** Technology, Liberalization, Complexity, Developing, Middle East, Economic development, Employment, Inequality, Macroeconomics, Public economics, Economics

## Abstract

Economic growth and development requires greater access to global markets, while developing countries face many challenges in terms of trade liberalization. That is why most of the countries relying on natural income sources have not been able to improve their indicators of economic complexity and high technology utilization. The purpose of this study was to investigate the impact of trade liberalization on the economic complexity as a strategy adopted by the Middle East developing economies during the period 2002–2017; using the panel vector auto regression model (PVAR). Immediate reaction test results show that, over a period of 10 years, economic complexity increases with positive shock from variables of trade freedom, foreign direct investment and gross fixed capital formation, but in the long run, the effect of imports of intermediate and capital goods is initially increasing and, after a short period, has a positive downward effect. In general, the results of this study recommend that; in order to achieve a proper share of export revenues in economic growth, the Middle East countries need to strengthen the foreign trade economy through trade liberalization and experience the impact of imports of medium and final capital goods, gross capital formation, and foreign direct investment in the index of economic complexity.

## 1 Introduction

The concept of economic complexity in a country refers to the production of domestically-based knowledge products as well as the diversification of export goods by the country. By economic complexity, the emphasis is on the intense application of technical knowledge in product diversification to encompass it in the domestic consumer markets on the one hand and foreign markets on the other. However, the economic complexity of countries' production is not limited to the ability to apply knowledge to the production process; rather it encompasses much broader dimensions (Utkovsklbi et al., 2018). Accordingly, the more diverse the country's export basket and the more sophisticated it is, the more powerful it is in terms of economic interactions at the international level and the more economically viable it is. The economic complexity was first discussed by Hidalgo, klinger, Barabassi and Hausmann (2007). They examined the networks of communication between products, or "product space” and the results showed that products with higher complexity rank were more interconnected in the middle region of the product space, while products with lower complexity rank were located in less interconnected areas. Simple and publicly used goods do not require much knowledge or, if they have, are produced by countries of low complexity, so they are less competitive internationally. As a result, the level of complexity of countries in commodity production can be determined by the index of economic complexity (Hidalgo et al., 2007). Further, Hidalgo (2015) stated, It is not only the extent to which every society enjoys production knowledge at the present time, but the extent to which the modern society at large is more committed to the use of aggregated knowledge. The criterion of the modernity of individual societies must be understood in terms of their ability to use collective and shared knowledge. To this end, it is necessary for individual societies to expand their activities in a network of active members, thereby enabling them to share their knowledge and common knowledge with the modern world. In other words, economic complexity is defined as the extent to which countries can produce and export knowledge goods through the knowledge formed in those countries (Lall et al., 2006). Since some products, such as computers and jet engines, can only be produced in complex societies, but commodities such as shirts and cereals can be produced almost everywhere; economic complexity is closely linked to the diversity of useful knowledge used. To create a complex and sustainable economy, societies with knowledge and technology must be able to interact with each other and combine their knowledge to produce products, so the concept of economic complexity is based on the combination of productive products of a given country and reflecting structures they have emerged for combining knowledge (Hidalgo and Hausmann, 2009).

Today, the role of science and technology in innovation and development is an important issue and the development of countries is based on solid science and knowledge that is the driving force behind the knowledge-based society (Moed, 2002). An economy that relies only on raw materials export will always be at risk of being trapped in a Dutch disease condition. For this reason, Zeufack (2002) relates the shift from the traditional economy to the modern economy to the changes in the structure of simple export products to complex and complex export products.

Generally, it can be stated that economic complexity is considered as an important and influential factor in the total production of the country that needs to be studied and determine its determinants (Felipe et al., 2012). By upgrading and strengthening the education sector to educate individuals, the government will increase the productivity of the workforce and the total productivity of the productive agents and give them a competitive advantage over other countries (Bournakis and Tsoukis, 2016). Therefore, in this economic approach, the most important determinant of a country's development is the amount of knowledge that is formed in that country, and the degree of knowledge of countries is directly related to the types of products produced there (Ferrarini and Scaramozzino, 2016; Bournakis and Tsoukis, 2016). In summary, what is certain is that the production of each product requires specific knowledge and the more diverse the productions of a country, the more integrated knowledge there presents.

Studies over the past decades have identified many factors in the improvement of the complexity of economic and international trade. Among the influential factors are the geographical situation, history, language and culture of the trading part countries. Eliminating the information constraints and transportation problems help to export competing products and automatically make the process of trading to geographically priority countries easier so that countries learn the right way to do business with their business neighbors (Jun et al., 2019). The presence of logical relationship between the types of products, to have sufficient knowledge about the geographical situation of trade neighboring countries, and having a common language and business boundaries, are all effective factors to increase business flow. Beside these factors, trade liberalization transfers knowledge and ideas, and private-sector firms can tap into the innovation of domestic production using high-tech product overflow, which increases their value-added. On the subject of globalization and trade liberalization, various definitions have been put forward by economists that believe the globalization of trade as a process of transformation that reduces political and economic boundaries, expands communications, enhances the interaction of cultures and pursues two fundamental goals. First, help to boost economic growth and employment by improving resource allocation and economic efficiency, and then help improve balance of payments by enhancing competitiveness of the export sector, diversifying and expanding export products, as well as making import substitute goods more efficient (Rudra and Tobin, 2017); Therefore, the positive and negative effects of trade liberalization on many economic variables, including economic complexity, are inevitable. The policy of trade liberalization should be seen as a social process in which the geographical constraints that are casting a shadow on social and cultural relations are becoming increasingly aware of the decline of these constraints. The globalization of business is the establishment of diverse and interconnected relationships between governments and societies that lead to the creation of the current global system in which decisions and actions in one part of the world can have important consequences for other people and communities (Ibrahim, 2015).

The contribution of the present study is to examine the impact of main factors on the economic complexity with emphasis on trade liberalization, as a strategy adopted by the Middle East developing economies during the period 2002–2017; using the panel vector auto regression model (PVAR). The empirical studies in this regard showed, countries that produce more sophisticated products in addition to diversification of production are usually more economically advanced and are expected to experience faster economic growth shortly. In this regard, the second part of the article is devoted to the subject literature and reviewing the empirical background. The final section also presents the conclusions of the study and finally policy proposals.

## 2 Theoretical background

From the economists' point of view, the technology and knowledge component has been interpreted as a factor in converting inputs into outputs, which creates a competitive advantage through value-added production (Porter, 1985). In a knowledge-based economy, high-tech services and industries play a key role, as it is a tool for technological excellence, creating competitive advantages and consequently increasing profitability. These industries have a growing share in the production of knowledge-based economies, and the share of low-tech and natural resources and raw materials industries has declined. Superior technology is the source of sustained export growth and the catalyst for sustained technological change and increased economic growth. Therefore, in knowledge-based economies, economic prosperity is created by providing the necessary platform for innovation and presence in global export markets. More and more entry into global markets requires the development of advanced industries, and the development of these knowledge-based industries requires the development of an innovation culture.

The important factor of knowledge is not only a byproduct of other older factors of production including labor force, capital, etc. but also a major source of production. The production and spread of science play an important role as the main driver of the countries' comprehensive and sustainable development. In fact, the production and spread of science has a profound impact on all economic, social and cultural spheres of the country (Drucker, 1998). On the other hand, scientific production and development play an important role as the main driver of the countries' comprehensive and sustainable development. In the present century, science and technology have been introduced as the most prominent elements of social life and political and economic power Therefore, the success of countries in the future will depend on how well they grow and their impact on their scientific, research, and strategic relationships. Economic complexity has the capability and tools needed to meet the challenges of economic growth and development as well as presenting new prospects for economic forecasting such as a dynamic forecasting system. For this reason, countries with economic complexity due to diversification use competitive advantage (Cristelli et al., 2015). As countries continue to advance in science, technology, and culture, they have been able to continually raise standards to the extent that they have mastered the world-wide markets and their new knowledge and technologies in national and transnational arenas. Applying knowledge and excellence in technology is one of the key indicators of community development (Koh et al., 2005). One of the indicators that made it possible to compare countries successfully in the knowledge-based economy and the productivity of all factors of production internationally is the use of the economic complexity approach. According to this approach, the amount of knowledge and productivity of all countries' production factors is directly correlated with the types of export products of that country (Hidalgo and Hausmann, 2009).

### 2.1 Complexity and development

One of the recent debates in economic development theory is the degree of diversity and expertise in countries' production and trade structure. From Adam Smith's theory of division and expertise in economic growth and development to the Hecker-Ohlin-Samuelson (HOS) international business model, the neoclassical economic position was that countries should specialize in comparative advantage in production and export, but then Since World War II, with the rebuilding of Europe and the independence of the former colonial countries, one of the first discussions on the development economy has been the discussion of diversification of production for economic growth and development, and for this reason governments have been involved in the process of industrialization and export diversification. The main idea of this debate is the result of Prebisch (1950) and the discussion of the severe pressure of Rosentine (1943). The main issue was the view that developing countries' dependence on the production and export of raw materials and raw materials makes them vulnerable to commodity shocks, price fluctuations, and exchange rates, due to low-income elasticity of demand for commodities.

In most economic growth models, science and technology play a central role and its development is the engine of economic growth. In the process of evaluating the socio-economic achievements of innovation in Europe, the United States, Japan, and some developed countries for the last decade, serious attention has been paid (Cozzens et al., 2002). Sustainable economic growth is largely explained by developments in science and technology and human capital. Research and development activities are one of the main sources of change in the production of knowledge and technology in a country. Accordingly, the level of knowledge of countries is directly related to the types of products produced in them. Production of any product requires specific knowledge. The more diverse a country's production means the more knowledge and accumulated knowledge there is (Sepehrdoust and Zamani Shabkaneh, 2018). In other words, the index of economic complexity can be used as a measure of a society's knowledge and skill level, and one may conclude that if a product requires a certain type of knowledge and skills, then countries that possess the knowledge and skills needed to produce high-tech products having developed economy (Bahar et al., 2014).

In the economics literature, growth and development has traditionally been measured by the macroeconomic variables such as GDP, while such averages cannot explain the increasing diversity associated with economic development alone. In the modern approach of new literature on economic development, the important measuring variable is the combination and variety of production products as well as the level of technological equipment used for economic growth (Gao et al., 2019). However, low-complexity economies have a poor accumulation of productive knowledge and less product diversity. As products from these countries are usually manufactured by many countries, they are referred to as inclusive products (Hausmann and Hidalgo, 2011). Similarly, pervasive ore publicly used products usually require lower capabilities to produce, while less pervasive and unique products require relatively more capabilities to produce. Accordingly, diversity and inclusivity are two important identifiers to identify the extent of a country's economic complexity for a product.

According to the structural models of economic development, countries must diversify their exports from primary products to factory products to achieve sustainable growth (Chenery, 1979). According to Prebisch (1950), this type of export diversification leads to a slowdown in the exchange relationship in countries that are dependent on exporting goods. Export earnings volatility is another important reason for diversifying exports that is similar to the portfolio effect. Since commodity prices often fluctuate, export-dependent countries suffer from volatility in export earnings, which can lead to risk aversion firms to reduce investment. As macroeconomic uncertainty increases, long-term economic growth is detrimental, so high-tech competitive product diversification and diversification of export products, in the long run, contribute to the stability of export earnings (Ghosh and Ostry, 1994). The theoretical basis for the diversification of export products based on the endogenous growth model is that the diversification of exports and the move from primary commodities to high-tech commodities leads to more growth because trade in such commodities leads to higher commodities. Productivity and effectiveness overflow (Herzer, 2006).

### 2.2 Complexity index

Complexity index theory is based on the premise that, products produced in an economy represent the amount of knowledge available, so productive knowledge can be equated with knowledge and skill. The index of economic complexity indicates the degree of complexity and variety of country's export product portfolio and is used to rank countries by level of complexity. Studies show, countries that have more than just a variety of products that also have complex manufacturing products are usually more economically advanced or expected to experience faster economic growth soon (Pugliese et al., 2014). The Economic Complexity Index (ECI), introduced by Hidalgo and Hausmann (2009) to measure the complexity of a country's economy, is based on the effective role of knowledge in explaining the differences in the level of per capita income and the rate of economic growth and development of countries. The index measures the success or failure of countries in the export of products and machinery and the ability to have a wider share in the production and export and trade of the world.

Following the introduction of the Index of Economic Complexity (IEC), experts like Tacchella et al. (2012), attempted to introduce a different measure of economic diversity comparable with IEC and stronger enough for exploring the relationship of more macroeconomic variables using a set of nonlinear iterative equations for exporting countries (Gao and Zhou, 2018). However, the use of the same linear regressions to measure countries' economic complexity index is not recommended for economies with different dominating regimes, which led economists to introduce new measurement indices. Studying the heterogeneous dynamics of economic complexity, Cristelli et al. (2015) observed that explanatory power of factors influencing economic development in countries with laminar regime is stronger than the explanatory power of these factors in countries with turbulent regime; therefore, the use of similar regression in dealing with heterogeneous economic situation of countries was considered inappropriate. Criticism on the shortcoming of ECI and Fitness metrics has led to introducing minimal extreme metric as a new form of fitness method that is claimed to be more appropriate for both chaotic regime and noise-free dataset. Among the recent studies are the Mariani et al. (2015) that attempted to study ECI and fitness comparative measurement ability and opened the way for providing a general form of “Fitness-Complexity” metric (Gao and Zhou, 2018).

While measuring economic complexity characteristics, two main characteristics of diversity and inclusiveness in a country's products are examined. The diversity characteristic of a country indicates how many products the country produces. Countries with high product diversification have a more complex economy. Products manufactured by few countries are more complex. By combining these two characteristics, it can be concluded that one country is in a better position than other countries in terms of productive education. Complex economies are capable of aggregating large amounts of productive knowledge across large networks of individuals and producing a diverse set of productive goods. In this view, the government can facilitate the production of high-tech goods and knowledge by adopting appropriate education and policy making to improve labor and capital productivity, thereby providing knowledge and diverse sophisticated skills move towards developing markets and guiding the economy toward the production of complex goods (Ben Hammouda et al., 2006). As technology affects the productivity of all factors of production, the government can increase labor and capital productivity by increasing the quality of public and civil services, improving the quality of regulations, and increasing the credibility of government commitment to policies.

### 2.3 Complexity and trade liberalization

Trade liberalization policy is an important factor in the growth of total productivity, and empirical studies have shown that the development and diversification of export and import trade-offs can help to increase total factor productivity and create trade freedom. Further, the competitive environment for the domestic industry is enhanced by the development of new production techniques or the efficient use of production factors, as well as the wider choice of lower quality intermediaries at lower prices for economic activity. Empirical evidences over the last two decades have drawn the attention of economists to the factors influencing the expansion of international trade. Experience has shown that having a common history, language and culture has led countries to show interest in expanding and diversifying trade with countries that already have extensive trade capabilities with the contracting country. In other words, the start for continuing bilateral trade on sophisticated and complex products is, to begin trade with those countries having a common history, language and culture on the one hand and a history of export and import with counterpart country on the other hand. For this purpose, countries need to use three measures for bilateral relatedness and trade with countries. The first emphasis is on identifying the type of product relatedness and finding out whether the exporting country had already export capabilities to the destination during the past periods? The second emphasis is on the relatedness of importing countries and whether the exporting country is trading the same product with neighboring countries of the destination? Lastly, the third concern relies on the relatedness of the exporter and to know whether the exporting country's neighbors had previously exported similar products to the destination (Jun et al., 2019).

The advantage of trade liberalization comes from the increasing competitive pressures of domestic and foreign firms on inefficient domestic firms to eliminate any waste of resources and to benefit from the economies of scale, thereby increasing the productivity of all factors of production in domestic firms. Trade liberalization, on the other hand, increases the capacity of the economy to attain more efficient production technologies and results in rapid productivity growth by increasing the import of intermediate goods and the transfer of knowledge and technology (Harrison, 1996). Expanding business relationships through economic freedom and the trade freedom channel is one of the most important goals of economic decision-makers worldwide. The most prestigious international institutions, Heritage Foundation, consider economic freedom as a measure by which individuals are free to produce, distribute and consumer goods and services. Freedom in practice is defined as non-coercion and restriction, which improves the progress of societies and improves the human condition. Thus, the signs of progress in freedom can be seen in the advancement of possibilities. The role of the state in providing such a free society, where all people can improve their lives in modern ways without the restrictions imposed by the governments is vital (Georgiou, 2015). Since freedom is defined as the absence of constraint, pressure, or constraint in choice of action, therefore economic freedom can be interpreted as the absence of any restriction on production, distribution, and consumption of goods and services.

In summary, it can be said that trade freedom leads to technology transfer through the import of advanced capital goods. Such imports of capital goods also boost export earnings growth and boost foreign capital inflows. According to the endogenous growth pattern, during the transition to trading freedom technology plays an important role in boosting exports as well as GDP growth. An increase in exports through the use of unused resources can lead to an increase in GDP. The increase in freedom-influenced imports also enables the purchase of foreign capital goods and improves technological progress. Finally, by focusing on the economic complexity and identifying the most important factors affecting it, we must pave the way for the emergence of capabilities and capabilities that are the same accumulated knowledge and skill. One of the less important factors, however, is economic variables, including trade freedom.

## 3 Experimental literature review

Various empirical studies have been accepted on the importance of trade freedom and its direct and indirect role in economic complexity. Most studies on economic complexity so far have focused on measuring and measuring this index or how it affects economic growth and examining factors affecting economic complexity such as the relationship between trade freedom and economic complexity. Therefore, the lack of attention to factors affecting economic complexity is observed.

Ferrarini and Scaramozzino (2016) and Jetter and Ramırez Hassan (2013) have emphasized that the optimal size of the government and its increased effectiveness can lead to the creation of a diverse and inclusive set of productive goods by enhancing the quality of services and long-term investment in education. The effectiveness of government fiscal and budgetary policies and its active presence in the economy can be attributed to the high quality of public services, effective planning and implementation of policies and the credibility of government commitment to policies. It provided the basis for the production of diverse and inclusive knowledge-based goods. The effectiveness and efficiency of the government are inseparable from the size of the state. Although the size of the state through financing through taxation and other sources of income provides the appropriate context for the creation of a business environment and leads to the production of highly sophisticated goods and specific knowledge, the size of the government cannot always have a positive impact on complexity. It is economical because if the government fails to spend its resources properly and scientifically on the parts that drive the production of knowledge, it will lead to a waste of revenue and a negative impact on economic complexity.

Studies by Bournakis and Tsoukis (2016) emphasized that the use of an economic complexity approach can lead to more rational and useful decision-making in the knowledge-based economy, therefore, the government must promote it about the knowledge-based components and the creation of appropriate contexts. In World Bank's definition, a knowledge-based economy is measured based on four components of science and technology, which include the components of institutional and economic regimes, innovation systems, education, and human resources, and information infrastructures, and is expected in developing countries by the government. Summary of studies on the topic of trade liberalization and economic complexity are presented in Table 1.

**Table 1:** Overview of the most important empirical studies related to the concept of economic complexity.

Title of the Research	Results and Findings
Jun et al. (2019) Bilateral relatedness: knowledge diffusion and the evolution of bilateral trade.	The results of the study showed that the emergence of a common language phenomenon with the fields of science and technology has made space for the exchange of more advanced and sophisticated products more diverse in the business world. Obviously, in the new approach of knowledge-based trading, attention is paid to the relatedness of exporting products, exporter and the product importer in a multilateral trading network.
Ebi and Eke (2018) Institutional Quality and Economic Diversification in Oil-Rich Economies: A Case Study of Nigeria, 1996–2016.	Using the error correction model, the results showed that despite a significant share of its exports, by strengthening the four indicators of government effectiveness, rule of law, political stability and corruption control, it was able to significantly diversify its GDP.
Gonzále et al. (2018) Multi-criteria analysis of economic complexity transition in emerging economies: The case of Paraguay. Socio-Economic Planning Sciences.	The research answers the general question of which manufacturing sector should be promoted through the transition to a more sophisticated economy to develop economically (according to the theory of economic complexity) in Paraguay? The results of these evaluations show that a combination of approaches can be beneficial, and with Paraguay, it helps identify areas that, if promoted by policymakers, can help economic development through the complexity and accumulation of economic potential.
Utkovski et al. (2018) Economic complexity unfolded interpret able model for the productive structure of economies; Emerging and developing countries, 2016–1970.	The results showed that countries that produce more diverse commodities are more capable of exporting more complex and diversified commodities, with few countries having the ability to export high-tech commodities. Possess and, with specific knowledge, export diverse and inclusive goods.
Zaccaria et al. (2015) A case study for a new metrics for economic complexity; The Netherlands, 1995–2001.	The results indicated that single-product segments produced high quality but low competitiveness. In contrast, the horticultural and energy sectors were highly competitive. They also scrutinized the pharmaceutical sector in greater detail, indicating a reduction in global complexity and a tendency to produce lower quality products.
Hartmann et al. (2017) Linking economic complexity, institutions, and income inequality; 122 countries including countries with a population greater than 1.5 million and total exports over one billion dollars, 1968–2008.	The result was that increased economic complexity was associated with reduced income inequality, and countries exporting complex products had lower income inequality than countries exporting simple products.
Gnangnon and Moser (2014) Intellectual Property Rights Protection and Export Diversification: The Application of Utility Model Laws; 89 Developing and Developing Countries, 1975–2003.	The results showed that the protection of intellectual property rights, commercial freedom, innovation, and an increase in GDP and human capital leads to diversification of export products and consequently economic complexity.
Pugliese et al. (2014) The discernment of heterogeneous country industrialization patterns through economic complexity; India, 1963–2012.	The results show that more complex and diverse economies when experiencing industrialization, fewer constraints on per capita GDP.
Jetter and Ramırez Hassan (2013) The Roots of Export Diversification; Selected Countries of the World, 1960–1960.	The results showed that out of the 43 factors examined, only four factors: natural resource costs as a percentage of GDP, primary school enrollment rate, population size and level of foreign direct investment are important in long-run export diversification.
Agosin et al. (2012) Determinants of Export Diversification around the world; Selected countries of the world, 1962–2000	The results show that the effect of distance to market variables and trade openness have negative effects on export diversification and the effect of education and human capital is positive and significant.
Parteka and Tamberi (2011) Export diversification and development - empirical assessment; 60 selected countries, 1985–2004.	The empirical findings show that relatively high expertise in economic structures is associated with low levels of per capita income, but countries diversify their export structures as they grow. However, usually only per capita income and, ultimately, country-specific fixed effects are the only explanatory variable that has been taken into account in estimating specialized curves. Moreover, geographical conditions of investment, human capital, distance from major markets and the size of the country are the most important and determining processes of the export diversification process.
Feenstra and Kee (2008) Export variety and country productivity: Estimating the monopolistic competition model with endogenous productivity; 48 OECD countries, 1980–2000.	In this study, the translog function has been used and the relative diversity of exports has a positive effect. Over the past two decades, the amount of export diversification has more than doubled. The overall increase in export diversification also increased productivity to 3.3 percent on average. The results of the estimation model show that 25% of OECD countries have intra-country productivity changes "equal to 31% and only a very small percentage of inter-country productivity changes".
Ben Hammouda et al. (2006) Diversification: towards a new paradigm for Africa's development; 1981–1983.	The results show that the effects of GDP formation, GDP, governance are positive and significant, and the impact of other variables such as GDP, GDP, trade openness, inflation, and negative exchange rates. Meaningful.

Based on the literature review and studies conducted on the relationship between trade liberalization and economic complexity and related government policies, which have been briefly explained in Table 1, this study aims to fill the gap on the subject of economic complexity and examines the impact of trade liberalization on the economic complexity as a strategy adopted by Middle East developing economies during the period 2002–2017; using the panel vector auto regression model (PVAR). The VAR model is a multi-equation system in which all variables are endogenously incorporated into the measurement model. One of the benefits of the VAR model is that it does not require any prior assumptions for a structural model and that the effects of variables on each other are allowed to estimate a structural model. If the time series dataset belongs to different segments, such as a set of countries, the PVAR method is used, which allows investigating the interaction between endogenous variables in the system (Olanrewaju et al., 2015).

## 4 Research methodology

Following the theoretical background and literature review of economic complexity elaborated by Ebi and Eke (2018), Gonzále et al. (2018), and Chen and Puttitanun (2005), the estimation model and the variables used for this study is presented as Eq. (1).

(1) ECI=F(TL,MI, FDI,GFCF)

Where, the dependent variable is the Economic Complexity Index (ECI), which indicates the degree of diversity of domestic production with the degree of differentiation or degree of overseas production that is intended to be a function of independent explanatory variables as; Trade Freedom (TL), Foreign Direct Investment (FDI), Import Of Intermediate And Capital Goods (MI) and formation of Gross Fixed Capital (GFCF).

### 4.1 Trade liberalization (TL)

One of the major factors affecting economic complexity is trade freedom, which is a composite index of the tariff and non-tariff constraints affecting exports and imports. Restrictions on the entry and exit of goods affect the production of high-tech goods, as trade freedom pressures on inefficient firms by increasing competition between domestic and foreign firms. Eliminate any waste of resources and enjoy economies of scale. As a result, total factor productivity in domestic firms increases. Now, if the conditions are provided for the country to move forward with the acquisition of new knowledge by enhancing the productivity of production factors, it will, therefore, leads to the production of sophisticated goods, high technology, and increased competitiveness. It can also be said that trade freedom by increasing the import of intermediate goods and the transfer of knowledge and technology increases the capacity of the economy to absorb superior technologies and provides the basis for the production of diversified and less inclusive goods (Harrison, 1996). It can, therefore, be said that the business model in which goods and services are traded between countries without government restrictions is called trade freedom, which has many economic benefits. On the other hand, imports are expected to bring in capital goods with new production technologies, and on the other hand, export orientation towards import substitution strategy and promotion of comparative advantage can increase national production. Improved competitiveness and enhanced economic complexity. Measures to measure and evaluate trade freedom include foreign trade tax, regulatory trade barriers, and the actual size of the trade sector compared to the expected size, the difference between the official and the black market rate, the control of international capital markets. In this study, data on trade freedom were extracted from the Fraser Institute statistical database.

### 4.2 Foreign direct investment (FDI)

Kojima (1977) views the foreign direct investment as a means of transferring technology, capital and knowledge from one country to another. Kojima believes that there are two types of foreign direct investment in parallel with foreign direct investment and anti-trade foreign direct investment. He believes that foreign direct investment will have a different impact on the host nation's trade, depending on whether it is trade-related or anti-trade. If foreign direct investment is in line with trade, it means that investing in industries where the investor country has a relative disadvantage will increase export growth. But if foreign direct investment has an anti-business orientation, it means that investing in industries in the host country where the investor country has no comparative advantage will not lead to export growth and growth; it can be understood that foreign direct investment provides the conditions for technology, capital and knowledge transfer and by acquiring knowledge from the foreign investor leads to the production of diverse and high-tech goods.

In the meantime, the World Bank has defined and directs foreign direct investment, meaning that foreign direct investment means having at least 2% of the voting shares in a profitable activity overseas by the foreign investor. It can be in the form of new investment, reinvestment, earnings and any contract between the parent company and the foreign company under the balance of payments (Gemmell et al., 2008). For the UN Research and Development Summit, FDI also means long-term economic relationships that represent the enduring benefits and control of an entity resident in one country (the parent company) over an entity resident in another country (sub-branch). Foreign direct investment is a process whereby a company directs and controls production activities in more than one country (Olise et al., 2013). Therefore, as a factor in the acquisition and transfer of knowledge, it provides the basis for enhancing the economic complexity of which statistics have been extracted from the World Bank statistical database.

### 4.3 Intermediate and capital imports (MI)

Knowledge-based growth models see economic growth as a result of research and development, which in turn affects a country's ability to achieve superior technology. R&D involves both internal and external categories and the development of innovation activities is subject to internal R&D activities. R&D is necessary, but developing countries devote a small share of their GDP to R&D activities, and we always see a significant gap between the innovation activities of developing countries with developed countries. But, based on extensive empirical studies, it can be stated that the innovation activities of countries are not only subject to domestic R&D activities but also to the overflow of other countries' R&D activities that can be transferred through the channel of importing capital goods and foreign direct investment intermediaries. When a country invests in research and development and improves its technology, neighboring countries can also benefit indirectly from the investment. The import of intermediate and capital goods is one of the factors that impede the knowledge of neighboring countries to the country, resulting in the acquisition of new science and technology and the production of diverse and high-tech goods. It is also worth mentioning that the mentioned index is equal to the ratio of total imports of intermediate and capital goods to total imports whose data are extracted from the World Bank statistical database.

### 4.4 Gross fixed capital formation (GFCF)

Capital assets such as buildings, machinery, and installations that are relatively long-lived and play a definite and sustainable role in the production process. By analyzing the relationship between the ratio of gross fixed capital in machinery and building to GDP and economic complexity, it can be said that gross fixed capital in machinery and building are determinants of productive production activities. These factors can provide the conditions for the production of high-tech goods and pave the way for the production of diversified and less comprehensive goods. But if machines and installations are not up-to-date with equipment and technology, it would not have such an impact on economic complexity. In addition to the skilled process of production, there is a need for high-tech machinery and capital, leading to manufacturing with state-of-the-art technology to increase the competitiveness of the product manufactured in the outside world. Data for this variable are extracted from the World Bank's statistical database. For this study, Eq. (2) is introduced and estimated; using as a panel vector auto regression model (PVAR) for the period 2002 to 2017 and the cross-sections of eight the Middle East developing countries. Besides, the logarithmic form of the data is used to homogenize the data.

(2) $$Log{\left(ECI\right)}_{it}=\beta_{\text{it}}+\overset{p}{\underset{j=1}{\sum }}\beta_{ecieci\;\;i_{,\;\;t-}\;j}\text{*}Log{\left(ECI_{i}\right)}_{t-j}+\overset{p}{\underset{j=1}{\sum }}\beta_{ecitl,\;\;\;i,t-j}\text{*}Log{\left(TLi\right)}_{t-j}+\overset{p}{\underset{j=1}{\sum }}\beta_{ecim\;i,\;t-j}\text{*}Log{{\left(MIi\right)}_{t}}_{-j}+\overset{p}{\underset{j=1}{\sum }}\beta_{ecifdi\;\;\;i,\;t-j}\text{*}Log{{\left(FDIi\right)}_{t}}_{-j}+\overset{p}{\underset{j=1}{\sum }}\beta_{ecigfcf\;\;\;i,\;t-j}\text{*}Log{{\left(GFCFi\right)}_{t}}_{-j}+\varepsilon_{it}$$

Where, Logarithm of Economic Complexity Index (Log ECI), is presented to be a function of independent explanatory variables as; Logarithm of Trade Freedom (Log TL), Foreign Direct Investment (Log FDI), Import of Intermediate and Capital Goods (Log MI) and formation of Gross Fixed Capital (Log GFCF). The subscript i: denotes the country, t: denotes for year and j: refers to p number of lags. The variables used for the estimation model of the study, the statistical sources of data for each variable and the predicted results before estimation are summarized in Table 2.

**Table 2:** Definition of variables used in the economic complexity model estimation of study.

Related Studies	Index	Symbol	variable
Studies by Bournakis and Tsoukis (2016), Jetter and Ramırez Hassan (2013), Parteka and Tamberi (2011) and few others have been conducted on this topic.	Evaluates the degree of diversity of domestic products with the degree of differentiation or degree of overseas products.	ECI	Economic Complexity
The results of the study Zaccaria et al. (2015), Gnangnon and Moser (2014), Agosin, et al. (2012), Pravin and Devashish (1998), and Harrison (1996) on this index acknowledge the following relationship: $\frac{\Delta ECI}{\Delta TL}∼0$	The criteria for measuring and assessing trade freedom include foreign trade tax, regulatory trade barriers, and the actual size of the trade sector compared to the expected size, the difference between the official rate and the black market rate, the control of international capital markets.	TL	trade freedom
According to the study by Vogiatzoglou (2009) about this index, the following relationship is expected: $\frac{\Delta \text{ECI}}{\Delta \text{FDI}}>0$	Foreign Direct Investment at Fixed Price 2010	FDI	Foreign Direct Investment
The results of the study of Parteka and Tamberi (2011), Agosin et al. (2012) and Ben Hammouda et al. (2006) on this index confirm the following relationship: $\frac{\Delta ECI}{\Delta \text{IM}}>0$	The ratio of total imports of intermediate and capital goods to total imports	IM	Share of intermediate and capital goods imports from total imports
The hypothesis regarding this variable is supposed to be as follows in most relevant studies. $\frac{\Delta ECI}{\Delta GFCF}>\;0$	The ratio of gross fixed capital in machinery and building to GDP	GFCF	Gross Fixed Capital formation

## 5 Results and findings

**Figure 1:**
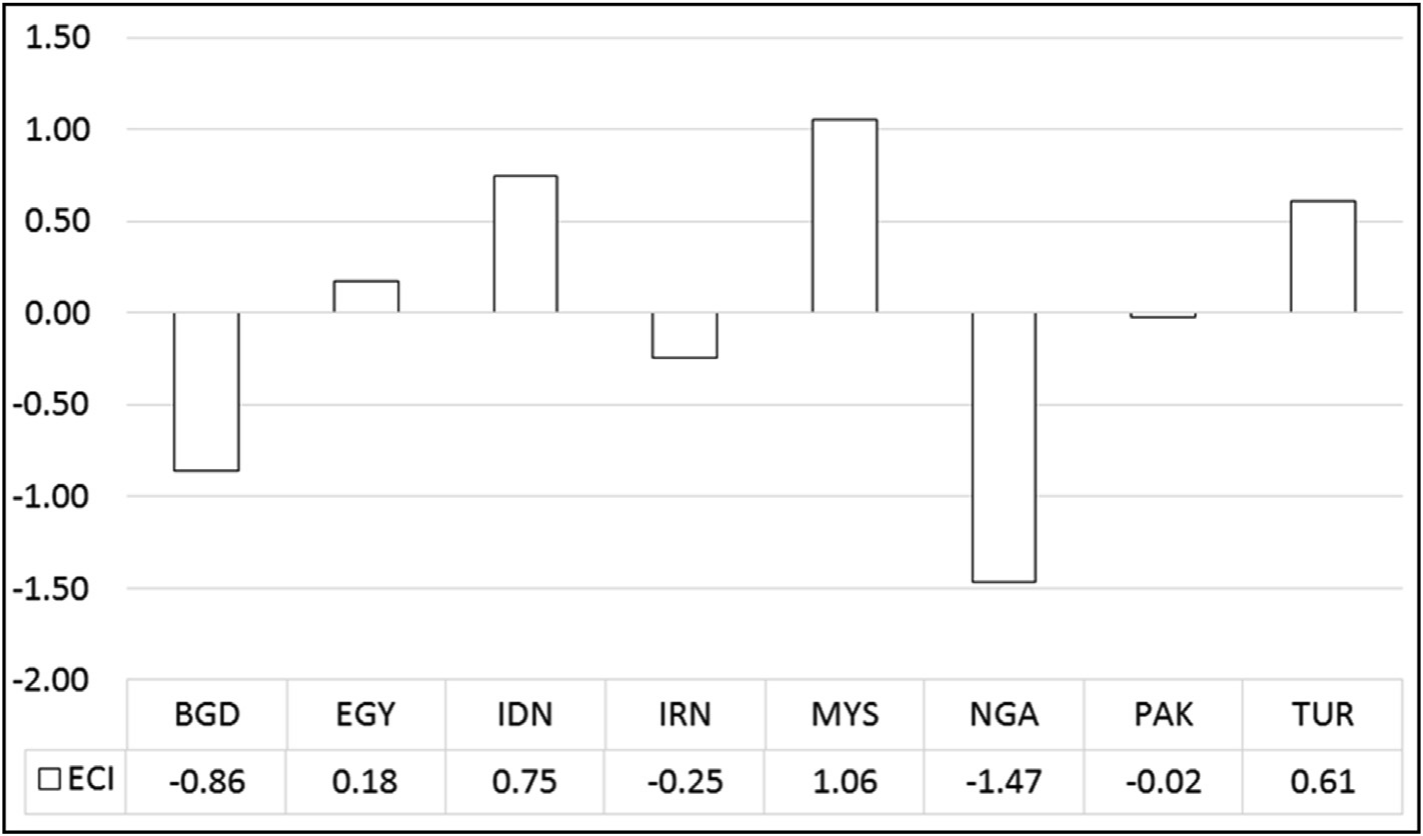
Average economic complexity of developing countries; 2002 to 2017. Source: www.atlas.media.mit.edu.

Before estimating the study model and analyzing it, the statistical analysis of trade freedom and economic complexity of the developing countries over the period 2002–2017 has been shown in Figure 1. The results show, the low economic complexity index in countries under study, with Malaysia and Nigeria having the highest and lowest economic complexity, respectively, with the mean of 1.06 and 1.47, respectively. Iran also averages -0.25.

**Figure 2:**
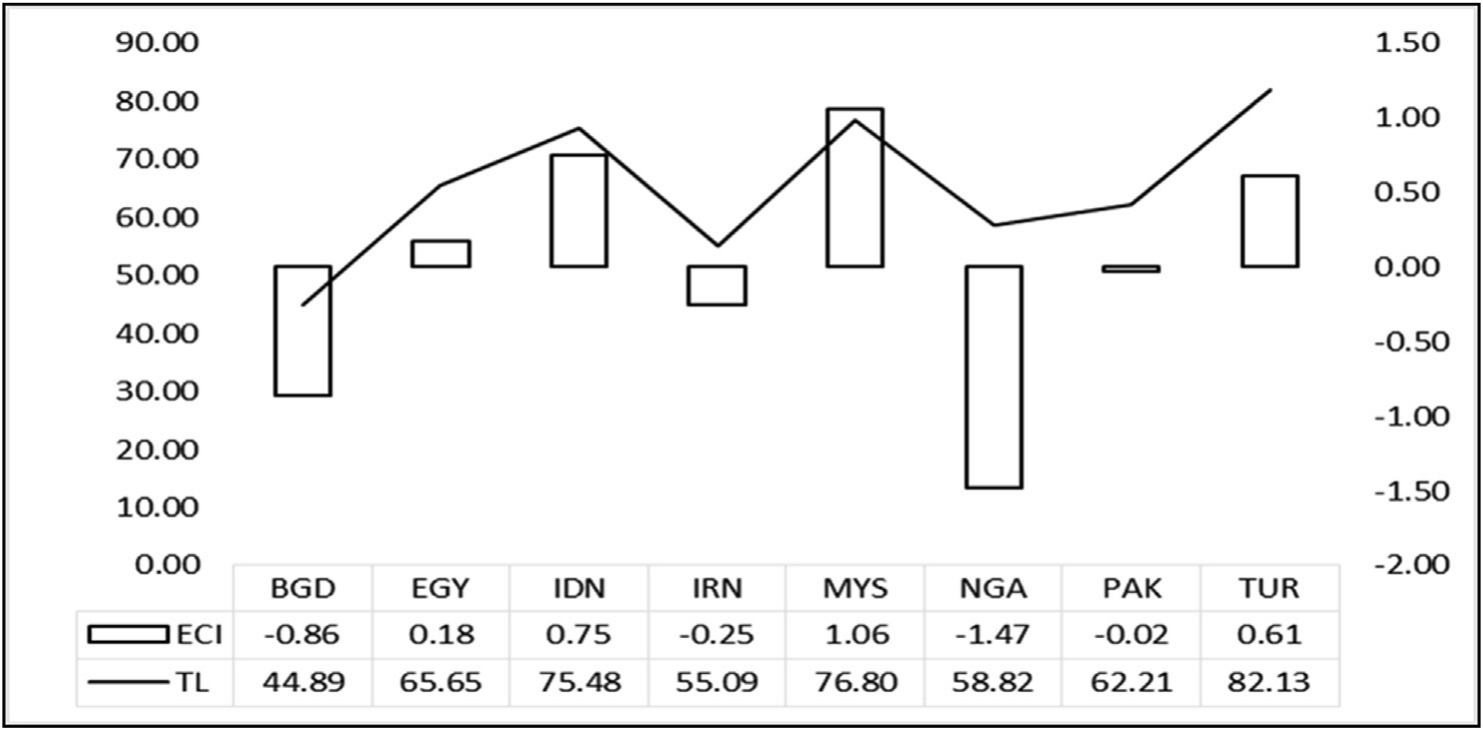
Comparison of the mean of trade freedom (TL) and economic. Complexity (ECI) Developing countries D8; 2002 to 2017.

Figure 2, shows the positive relationship between trade freedom and the economic complexity of developing countries during the period of 2002–2012. Both countries, Nigeria and Bangladesh are less economically complex during the average period under study, with lower levels of trade freedom than Malaysia.

Before any further estimating process, the Levin, Lane, and Chow Unit Root Test (LLC) were used to investigate the stability of variables over time. The results of this test showed that all variables are stationary at level and no co-integration test is required. Further, the optimal lag of the model was determined for estimating the vector autoregressive model and controlling the degree of freedom based on the Schwarz Bayesian criterion. The results in Table 3 show that the maximum optimal lag is 1 (star).

**Table 3:** Optimal lag determination.

HQ	SC	AIC	lag
*-7.012	*-6.398	-7.41	1
-6.251	-5.126	-6.982	2
-5.777	-4.142	-6.84	3

**Figure 3:**
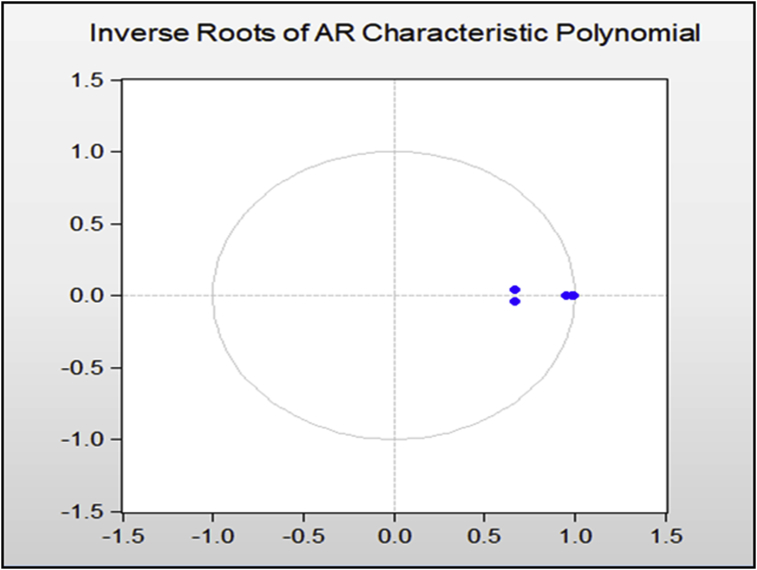
Model Stability Test (Source: Research findings).

To estimate the panel vector auto-regressive model (PVAR), it is necessary to evaluate the stability of the model. As we know, the estimated VAR is stable (stationary) if Eigen values have modulus less than one and centered inside the unit circle. The stability test results of the model in Figure 3, show that since all roots of the model are less than one and the root matrix dots are enclosed within a single circle; therefore the stability condition (PVAR) is established and the conditions for estimating the panel vector autoregressive model (PVAR) is fulfilled (Figure 3).

### 5.1 Analysis of impulse response functions

**Figure 4:**
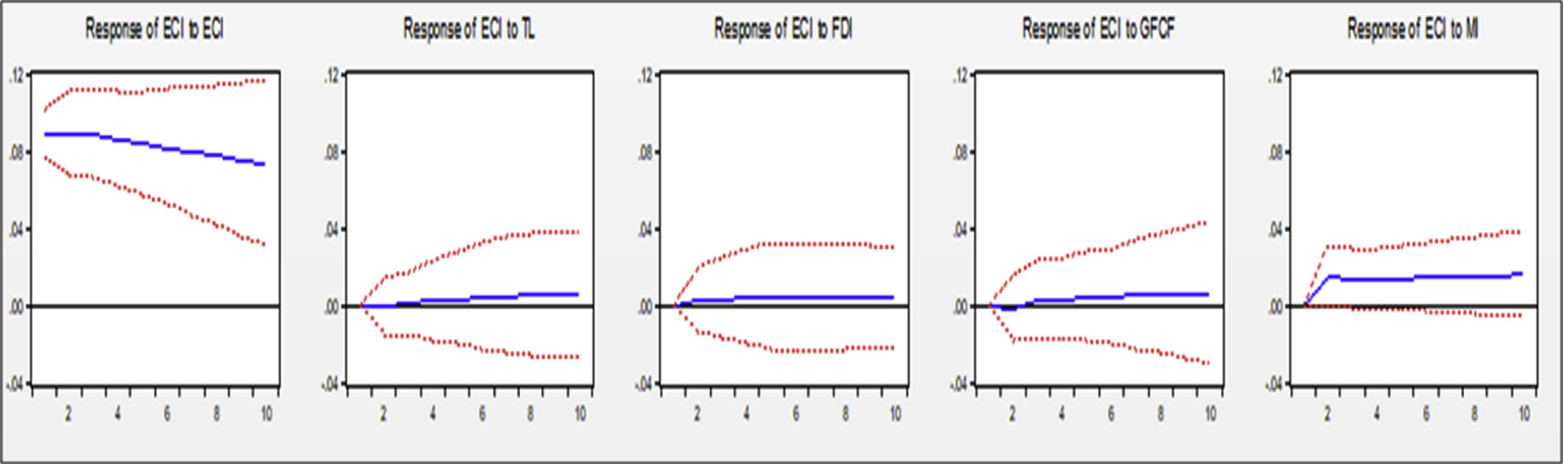
Complex economic response to shocks caused by explanatory variables (Source: Research Findings).

One of the applications of the PVAR model is to investigate the response of the model variables to the shocks in each of the variables and the estimated coefficients in the model have no specific economic interpretation. However, the results of the impulse response function can contain important interpretations. To this end, the present study examines the response of economic complexity to trade freedom of developing countries. Investigating the shock effect of the variables included in the model on economic complexity shows how much of an impact a shock would have on the economic complexity over different periods if a shock (shock) occurs. The results of the impulse response function are shown in Figure 4, which discusses the response of economic complexity to shocks introduced by explanatory variables.

#### 5.1.1 Economic complexity reaction to economic complexity shock

The economic complexity response is a positive economic complexity shock that does not tend to zero after 10 years period, so a positive shock to the economic complexity increases the economic complexity. Based on the Figure 4, the effect of this shock is long-term, so the results indicate that the production of diverse and distinctive goods within the country and the diversification of export products can for a long time lead to increased economic complexity in developing countries.

#### 5.1.2 The economic complexity reaction to the shock of trade freedom

Over 10 years, creating a positive shock to trade freedom promotes economic complexity. As shown in the graph, the increase in trade freedom initially had little positive effect on economic complexity, but as the effect of increased trade freedom on economic complexity increased, it eventually increased to about 10 years after its effect is still increasing. Therefore, trade freedom can be considered as one of the factors affecting economic complexity that affects the production of diverse and distinct goods by limiting entry and exit, as trade freedom creates the ground for acquiring new knowledge resulting in the production of sophisticated goods, with superior technology and increasing the competitiveness of countries. Business freedom can also, over time, enhance the capacity of the economy to absorb superior technologies and provide the basis for the production of high-tech goods through the increased import of intermediate goods and the transfer of knowledge and technology. The results of studies by Zaccaria et al. (2015); Gnangnon and Moser (2014); Agosin et al. (2012); Pravin and Devashish (1998), and Harrison (1996) are in line with the results of this study.

#### 5.1.3 The reaction of economic complexity to foreign direct investment shock

Over 10 years, creating a positive shock in FDI increases economic complexity. As shown in Figure 4, the increase in foreign direct investment initially has a little positive effect on economic complexity, but as the effect of increased foreign direct investment on economic complexity increases, it gradually increases until eventually, after about 10 years, its effect is still increasing. The foreign direct investment variable has a similar situation to commercial freedom over economic complexity. It is observed that the trend of foreign direct investment impacts on long-term and positive economic complexity. Foreign direct investment can provide the conditions for moving towards ICT exports, thereby increasing the knowledge and technology of developed countries by increasing the export of ICTs over time. The results of Vogiatzoglou (2009) studies are in line with the results of this study.

#### 5.1.4 The reaction of economic complexity to shock of gross fixed capital

Over 10 years, creating a positive shock to the formation of gross fixed capital raises economic complexity. As shown in the graph, the increase in gross fixed capital formation initially had a small positive effect on economic complexity, but as the effect of increasing gross fixed capital formation on economic complexity increased, it gradually increased until finally after about 10 years. It's still incremental. The variable gross fixed capital formation of high-tech commodities provides the initial capital and the acquisition of new knowledge and technology results in the production of diversified and less comprehensive commodities.

#### 5.1.5 The reaction of economic complexity to the shock of imports of intermediate and capital goods

In 10 years period, creating a positive shock in the import of intermediate and capital goods would initially increase the economic complexity, but these effects would not be permanent, and after about three years, the effect would be incrementally reduced. As the graph shows, its impact is long-lasting, and after 10 years its positive impact on economic complexity remains. The import of high-tech goods transfers’ knowledge from the importing country to the developing countries, and manufacturers can focus on the technology of importing intermediate and capital goods to produce diversified and inclusive goods and increase economic complexity. Concerning the aforementioned variable, the results of the study are consistent with the study of Parteka and Tamberi (2011).

### 5.2 Analysis of variance of prediction error

The contribution of each variable in the model is determined by their changes over time and for that, the analysis of variance of prediction error was used in this study. The purpose of the analysis of variance analysis is to determine the relative contribution and significance of impulse induced by each variable in its changes relative to changes in other variables. The results of the analysis of variance of the prediction error for the variables studied over six years are presented in Table 4. The results show that, in the first period, a hundred percent of the variance in economic complexity was explained by itself and the contribution of other explanatory variables was zero. According to the results from the second to tenth period, the share of economic complexity variable decreased from 98.605% to 96.778%, which has the highest share in explaining economic complexity among the explanatory variables of the model. Commercial freedom also rose from 0.014% to 0.17% from the second to the tenth period.

**Table 4:** Analysis of variance of prediction error for the 3 years.

Period	SE	L(ECI)	L(TL)	L(FDI)	L(MI)	L(GFCF)
1	0.088	100.000	0.000	0.000	0.000	0.000
2	0.126	98.605	0.014	0.019	1.333	0.037
3	0.154	98.299	0.009	0.047	1.601	0.042
4	0.177	98.063	0.017	0.087	1.771	0.058
5	0.196	97.844	0.038	0.112	1.992	0.082
6	0.213	97.635	0.064	0.129	2.061	0.109
7	0.227	97.425	0.092	0.143	2.201	0.136
8	0.241	97.214	0.120	0.154	2.346	0.164
9	0.253	97.001	0.146	0.163	2.497	0.191
10	0.263	96.785	0.170	0.17	2.655	0.217

The results in Table 4 for the FDI variable show that in the second period, the share reached 0.019%–0.17% in the tenth period. Besides, the variable share of imports of intermediary and capital goods from the second to the tenth period ranged from 1.323% to 2.655%. Also, the variable share of gross fixed capital formation from the second to the tenth period has ranged from 0.037% to 0.0217%; thus, it is considered in line with Table 4 that in the long run over a period (10 years) about 96% of the changes in economic complexity is explained by the economic complexity itself, each of the variables of trade freedom and foreign direct investment explain approximately 0.17% of the volatility, and the intermediate and capital imports variable indicate near 2.5% of the fluctuates in economic complexity. The share of gross fixed capital formation in fluctuations is equal to 0.217%, therefore the results of the analysis of variance as instantaneous reaction functions show the long-run effect of explanatory variables on economic complexity.

## 6 Conclusions and recommendations

Considering the factors affecting economic complexity is one of the essentials in achieving the goal that commercial liberalization is one of the important factors affecting economic complexity and because of ignoring these effective factors, this study examined the impact of policy using the panel vector auto regression model (PVAR). The government's commercial freedom focuses on the economic complexity of the Middle East developing countries during the period 2017–22. The economic complexity with the production of diversified and less inclusive goods increases the production of productive goods to create new job opportunities and thus leads to economic growth and development. By upgrading knowledge and integrating it, we can produce more sophisticated goods, and to achieve this stage we must seek new knowledge and superior technology to take this step. Therefore, it is necessary to move in the direction of increasing economic complexity so that the employment and welfare of the general public can be achieved to achieve the goal of economic growth. Countries whose governments have pursued sound and scientific policies to improve the productivity of all factors of production and production of specific knowledge have been able to create more favorable conditions for the production of high-tech and sophisticated goods, and the power of competition. Increase their receptivity.

Most developing countries face problems such as low levels of per capita income and low rates of economic growth. These countries have always suffered from a low level of per capita income and a widening income gap. Therefore, to overcome such problems, they require sustained and sustained economic growth but face different constraints and solutions for achieving economic growth. One of the solutions for which they have had many successes is the reliance on the production of knowledge-based products as well as the diversification of export goods. It can be said that eradicating poverty and adjusting income inequality, when considered with economic growth, become the biggest goal and the most difficult task of economic policymakers in the aforementioned countries. Based on the similar economic structure among the Middle East developing countries and given their common goal of social justice, reducing the class gap and achieving economic growth and prosperity, studies of the economic complexity of these countries are necessary.

The results of the study show that, over a 10-yearly period, creating a shock to commercial freedom has a positive impact on economic complexity. Initially, its positive impact on economic complexity is negligible, but as the impact of increased trade freedom on economic complexity increases, as trade freedom reduces the entry and exit restrictions on the production of high-tech goods and through increased imports. The intermediary goods and the transfer of knowledge and technology increase the capacity of the economy to attract the highest technologies and provide the basis for the production of diversified and less inclusive goods. Also, a positive shock in the import of intermediate and capital goods initially led to an increasing increase in economic complexity but these effects were not permanent and after about 3 years the effect was gradually decreasing.

The import of high-tech commodities causes the knowledge to be transferred from developed countries to the developing countries, and the producers move with the technology of importing intermediate goods and capital to produce high-tech commodities. Gross fixed capital formation (FDI) and foreign direct investment (FDI) are similar to the effects of free trade on economic complexity and the effect of gross fixed capital formation and FDI is positive. Despite the Middle East developing countries' position in economic complexity, these weaknesses are still seen in these countries as not being able to move sufficiently to produce high-tech, competitive export commodities. According to the results of the study, it is recommended that developing countries take steps to improve the index of economic complexity to reduce restrictions on entry and exit of high technology goods in the form of free trade with mutually beneficial goals. To this end, it is necessary to create the right conditions for attracting foreign direct investment, upgrading infrastructure and appropriate infrastructure, enjoying the global standard, as well as removing investment barriers to support development projects in production and the export of goods and services in the ICT sector.

## Declarations

### Author contribution statement

Hamid Sepehrdoust, Razieh Davarikish, Maryam Setarehie: Conceived and designed the experiments; Performed the experiments; Analyzed and interpreted the data; Contributed reagents, materials, analysis tools or data; Wrote the paper.

### Funding statement

This research did not receive any specific grant from funding agencies in the public, commercial, or not-for-profit sectors.

### Competing interest statement

The authors declare no conflict of interest.

### Additional information

No additional information is available for this paper.
